# Topological deep learning for drug–target interaction, virtual screening, and docking scoring: a practical, benchmark-driven review

**DOI:** 10.1093/bib/bbag370

**Published:** 2026-07-12

**Authors:** Beatriz Suay-García, Antonio Falcó

**Affiliations:** Departamento de Matemáticas, Física y Ciencias Tecnológicas, Universidad Cardenal Herrera-CEU, CEU Universities, C/ Luis Vives, nº 2 (46115) en Alfara del Patriarca, Valencia, Spain; Departamento de Matemáticas, Física y Ciencias Tecnológicas, Universidad Cardenal Herrera-CEU, CEU Universities, C/ Luis Vives, nº 2 (46115) en Alfara del Patriarca, Valencia, Spain

**Keywords:** topological deep learning, persistent homology, drug–target interaction, virtual screening, docking scoring, benchmarks

## Abstract

Artificial intelligence is now central to computational drug discovery, yet performance in core tasks—drug–target interaction (DTI) prediction, virtual screening (VS), and docking scoring—is still limited by the multiscale geometric nature of molecular recognition and by evaluation pitfalls such as dataset bias and leakage. Topological deep learning (TDL) offers a complementary route to encode global and multiscale structure from ligands, binding pockets, surfaces, and protein–ligand complexes via persistent homology and related constructions. This review provides a practical, task-driven synthesis of TDL methods for DTI/VS/docking scoring, with an emphasis on design choices that determine real-world utility: (i) data modality (ligand, pocket, or complex/pose) under controllable uncertainty, (ii) topological objects and filtration families (distance/alpha versus physicochemical or interaction-field filtrations), and (iii) vectorizations and integration patterns (persistent homology-as-features, hybrid geometric deep learning, and emerging end-to-end approaches). Distinct from prior surveys, we present a decision-oriented taxonomy and a benchmark-driven evaluation playbook that specifies minimum standards for splits (scaffold, temporal, and target-wise/cluster), metrics (including early-recognition metrics for VS), baselines, and ablations to isolate the topological contribution. To support reproducibility, we provide a reporting checklist and curated summary tables (methods matrix and benchmark recommendations) that map tasks to recommended protocols and common failure modes.

## Introduction

### Motivation and scope

Artificial intelligence (AI) has become central to computational drug discovery, enabling models that scale across large chemical libraries, biological targets, and heterogeneous assay readouts. Yet key tasks—drug–target interaction (DTI) prediction, virtual screening (VS), and docking scoring—remain limited by a shared bottleneck: molecular recognition is driven by *multiscale 3D geometry*. Binding depends on ligand shape, binding-site architecture, and interface structure that are only partially captured by common featurizations (fingerprints, local message passing on 2D graphs, or voxelized fields). As a result, deep learning (DL) systems can generalize poorly under distribution shift (new targets or chemotypes) and often provide limited mechanistic insight.

Topological deep learning (TDL) addresses this gap by making *global* and *multiscale* structure accessible to learning. Persistent homology (PH) provides a principled route to summarize geometry as stable multiscale signatures, and early work demonstrated its utility for biomolecular prediction [1]. The paradigm has since broadened: topology can appear as (i) PH-derived descriptors, (ii) topology-informed regularization, or (iii) architectural priors that shape representation learning [2, 3].

This review targets practitioners working on DTI, VS, and docking scoring who need *actionable* guidance: which modality to use (ligand/target/pocket/complex), how to construct topology (object + filtration), how to integrate it (vectorization + fusion), and how to evaluate fairly (benchmarks, splits, metrics).

**Scope boundaries.** We focus on DTI, large-scale screening/ranking, and docking-related scoring because they sit at the high-throughput core of modern discovery and expose the dominant challenges topology is meant to address (geometry, sparsity/noise, and generalization). We only touch on generative design when it motivates reusable methodology, and we avoid a full mathematical treatment in favor of the minimum concepts needed to implement and evaluate TDL workflows reliably.

### Why topology in drug discovery now

Topology is most useful when the *shape of data* matters as much as the values attached to it. PH summarizes multiscale structure—components, loops, and cavities—by tracking how topological invariants evolve along a filtration [4–6]. In molecular settings, this is directly relevant: ligands and binding sites exhibit hierarchical organization (local neighborhoods, mesoscopic motifs, channels, and pockets) that jointly drive affinity and selectivity.

PH-based representations can be robust to modest geometric variability when constructions are controlled, and they can yield interpretable multiscale signatures that map back to structural motifs. These motivations underlie early topological representations for molecular machine learning (ML) [7] and topology-enhanced predictors for binding endpoints [8, 9]. Recent work increasingly treats topology as a complementary signal within multimodal pipelines (e.g. combining topology with graph/sequence or language-model embeddings) [10, 11]. Representative recent advances include TopoFormer, which converts 3D protein–ligand complexes into natural language processing-compatible sequences via a persistent topological hyperdigraph Laplacian (PTHL), achieving state-of-the-art performance across scoring, ranking, and screening benchmarks [12]; CAML, which applies commutative-algebra representations to protein–ligand complexes and improves binding affinity prediction on PDBbind and CASF benchmarks [13]; and mGLI, which extends knot-theory tools to a multiscale Gauss link integral framework competitive with top-ranked methods across 13 biological datasets including protein–ligand interactions and toxicity screening [14].

As the field matures, *evaluation rigor* often becomes the limiting factor. Diagram vectorizations trade stability, expressivity, and computational cost, and drug-discovery benchmarks are particularly sensitive to bias and leakage [15]. Accordingly, topology is impactful only when paired with disciplined protocols, which this review makes explicit.

### How this review differs from existing surveys

Several surveys cover topological machine learning (TML/TDL) broadly [2, 3], and recent domain reviews discuss topology in AI-driven drug discovery [16]. Our contribution is narrower and operational:

- **Task-first organization.** We center *DTI, VS, and docking scoring*, mapping each task to suitable topological constructions, integration patterns, and common failure modes.
- **Benchmark-driven guidance.** We provide an evaluation playbook—benchmarks, split protocols, metrics, minimum baselines, and ablations—to reduce overoptimistic claims driven by leakage or incomparable setups.
- **Decision-oriented synthesis.** We distill design choices into a decision tree and a methods matrix aligned with data availability (2D versus 3D; pocket/pose availability) and compute constraints.

### How to use this review

**Figure 1:**
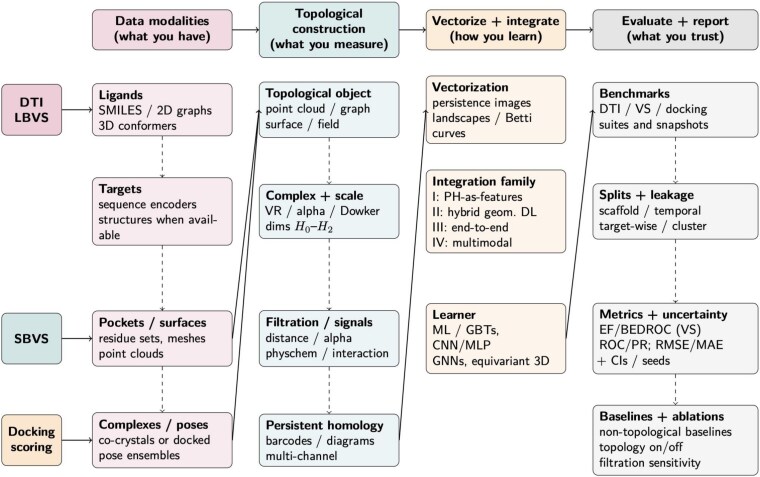
End-to-end TDL pipeline for DTI, VS, and docking scoring.

**Figure 2:**
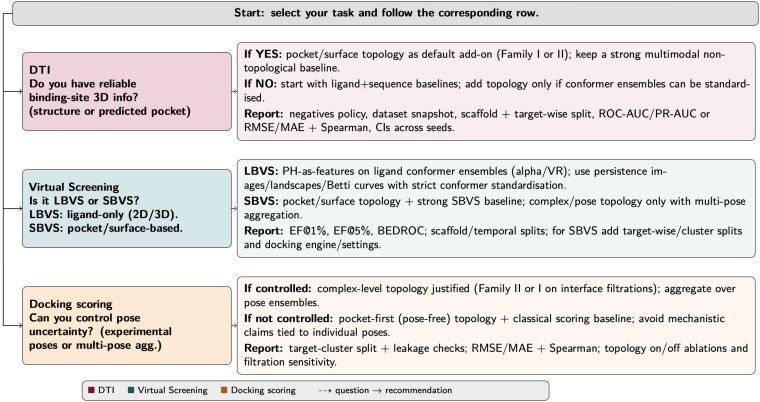
Decision tree for selecting a TDL strategy in DTI, VS, and docking scoring.

Start with the end-to-end pipeline schematic in Fig. 1, which maps the workflow from data modality to topological construction, vectorization/integration, and evaluation. Then use the decision workflow in Fig. 2, which routes DTI/VS/docking use cases to appropriate TDL families based on task formulation, 3D availability, and compute constraints. Table 1 summarizes representative methods (construction, integration strategy, and validation setup). Finally, Section Benchmarks, evaluation protocols, and reproducibility (see also Table 2) provides an evaluation playbook (benchmarks, leakage-resistant splits, metrics, baselines, and minimum reporting standards) to support fair comparison and reproducibility.

**Table 1:** Methods matrix for TDL in DTI, VS, and docking scoring. Rows summarize representative papers and the minimum information needed to reproduce the topological pipeline (representation $\rightarrow$ filtration/signal $\rightarrow$ vectorization $\rightarrow$ learner).

Task	Representative method	Family	Topological object	Filtration/signals	Vectorization/learner	Code
DTI	**Top-DTI** [10]	I	Ligand–protein contact graphs/complexes	Distance/ interaction-based filtrations; task-specific signals	PH summaries as features + classifier/regressor; DTI-focused design	—
VS (ligand-based)	**PH VS** [17]	I	Ligand 3D conformers as point clouds	2-parameter filtrations (shape + physicochemical fields); multi-parameter persistence	Fibered barcodes / Hilbert functions; similarity metrics (matching distance / $\ell ^{2}$) for kNN ranking	—
VS (ligand-based)	**ToDD** [18]	I/II	Substructure graphs/multiparameter signatures	Multi-parameter persistence built from chemistry-informed partitions	Topological fingerprints + metric learning (triplet/contrastive) for ranking	—
Docking scoring/affinity	**TopologyNet (TNet-BP)** [1]	II	Element-specific atom clouds for complexes	ESPH / multicomponent filtrations; interaction-scale signals	PI-like channels + CNN/MLP scoring	—
Docking scoring/VS	**Representability of algebraic topology** [19]	I/II	Complex-level element-specific clouds	ESPH + multiscale interaction radii	Topological descriptors + ML / CNN scoring; early structure-based VS use cases	—
Docking scoring / affinity	**ESPH + ML** [8]	I	Protein–ligand complexes (element-specific)	Distance filtrations; hydrophobic / electrostatic stratification (ESPH)	Vectorized persistence summaries + tree models / shallow nets	—
Docking/pose + ranking	**MathDL (GC2/GC3)** [20]	II	Algebraic graphs + topological fingerprints	Multiscale graphs + ESPH features across interaction cutoffs	Multichannel features + CNN / deep ensembles for pose + affinity ranking	—
Docking/pose + ranking	**MathDL (GC4)** [9]	II	Algebraic graphs + topological fingerprints	As above; task-tuned interaction features	CNN / GAN components for pose; CNN/regression for affinity / ranking	—
Docking scoring/affinity	**PATH** [21]	I/II	Complex-level atom clouds / interaction fields	PH over interaction geometry; selected interpretable fingerprints	Persistence fingerprint + gradient-boosted regression trees; includes binder/non-binder scoring	—
Docking scoring/affinity	**HPC** [22]	I	Hypergraph representation of protein–ligand interactions	Filtration over hyperedges; cohomology-based invariants	Persistent cohomology descriptors + ML (e.g. GBT)	—
Docking scoring/affinity	**PSH-ML** [23]	I	Nested hypergraphs; Hodge Laplacians	Spectral persistence over filtration scales	Spectral-persistence fingerprints + GBT for affinity	—
Docking scoring/affinity	**PerSpect ML** [24]	I	Simplicial / spectral constructions for complexes	Persistent spectra across multiscale interaction graphs	Persistent spectral features + ML regressors	—
Docking scoring/affinity	**DCML** [25]	I	Dowker complexes from bipartite interaction relations	Filtration over relation thresholds	Dowker-complex descriptors + ML for affinity	—
Docking scoring/affinity	**PMH / Mayer-homology learning** [26]	I	Persistent Mayer homology features on complexes	Generalized persistence ($d^{N}=0$) across scales	Multiscale topological vectorizations + ML regressors	—
Docking scoring/affinity	**PDFL** [27]	I	Directed flag complexes / directed interaction graphs	Persistent directed flag Laplacian across thresholds	Spectral topological features + multi-kernel ML for affinity	—
Docking scoring/affinity	**TopoFormer** [12]	II/III	Protein–ligand complexes converted to topological sequences via PTHL	PTHL; multiscale 3D-to-1D conversion across interaction scales	Topological invariant sequences + transformer (MAE self-supervised pretraining + supervised fine-tuning); scoring, ranking, docking, and screening tasks	GitHub
Docking scoring/affinity	**CAML** [13]	I/II	Protein–ligand complexes represented as commutative algebra objects	Commutative-algebra-based filtrations; algebraic topology combined with spectral theory	Commutative-algebra descriptors + ML regressors; evaluated on PDBbind and CASF benchmarks	—
Docking scoring/affinity	**mGLI / KDA** [14]	I	Atomic curve segments and knot-like structures in protein–ligand complexes	Multiscale Gauss link integral; element-specific and atom-specific variants across radius scales	mGLI features + gradient-boosting or deep neural networks; validated on 13 biological datasets including protein–ligand affinity, hERG screening, and toxicity	GitHub

*Note (Code column):* “—” indicates no public repository was identified at the time of writing; linked entries point to known public repositories.

**Table 2:** Benchmarking playbook (recommended default protocols) for DTI, VS, and docking scoring.

Task	Recommended datasets	Recommended split(s)	Core metrics	Common pitfalls
DTI	Davis; KIBA; BindingDB/ChEMBL curated subsets (optionally via TDC task definitions)	Scaffold split + protein-family/target-wise split; temporal split when timestamps exist	Classification: ROC-AUC, PR-AUC; Regression: RMSE/MAE + Spearman (report CIs / across-seed variability)	Negatives policy; assay heterogeneity/unit mismatch; duplicate pairs across splits; target leakage via close homologs
VS	DUD-E; DEKOIS 2.0; LIT-PCBA (report exact preprocessing and redundancy removal)	Scaffold split (ligand generalization); temporal split (when available); target-wise/target-cluster split for SBVS	Early recognition: EF@1%, EF@5%, BEDROC (optionally ROC-AUC as secondary)	Decoy/analog bias; redundancy/leakage (including across targets); metric misuse (ROC-AUC alone); over-tuning on benchmark idiosyncrasies
Docking scoring	PDBbind (general/refined + core); CASF-2016 benchmark suite (specify pose generation)	Target-cluster split (default) + leakage controls for homologs; optionally scaffold split for ligands; cross-docking sensitivity when feasible	Affinity: RMSE/MAE + Spearman (and ranking where relevant); Pose selection (if included): top-$1$/top-$N$ RMSD success	Target/pocket leakage; pose-generation leakage; dependence on docking engine/settings; mixing scoring versus pose-selection claims without protocol clarity

## Data modalities and topological representations for DTI/VS/docking

### Core data modalities in DTI, VS, and docking scoring

DTI prediction, VS, and docking scoring require representations of (i) the *ligand*, (ii) the *target* (typically a protein), and, in structure-based settings, (iii) the *binding site* and/or the *ligand–target complex*. TDL design therefore starts by stating what information is available (2D versus 3D; pocket versus pose) and what uncertainty is controllable.

**Ligands.** Ligands are commonly represented as SMILES strings [28] or 2D graphs. Classical ligand-based screening uses circular fingerprints (e.g. ECFP) because they are fast and competitive for similarity and activity modeling [29]. Deep baselines learn directly on graphs via message passing and related graph neural networks (GNNs) [30]. Topology is most informative when a reliable 3D embedding exists: conformer ensembles, atom point clouds with physicochemical channels, or weighted distance graphs. PH-based summaries can then capture multiscale geometric patterns and can be stable to small coordinate perturbations when constructions are controlled [7].

**Targets, pockets, and surfaces.** Targets can be encoded from sequence (k-mers, learned embeddings, protein language models) or from 3D structure when available. Structure-aware pipelines often localize the input to a binding pocket (atoms/residues within a radius of a reference ligand) or to an explicit pocket surface/shape representation. These modalities are natural candidates for topological summaries because pocket geometry exhibits multiscale cavities and channels that are not purely local.

**Complexes and poses.** Docking scoring adds pose uncertainty: models may score a single pose, multiple poses per ligand, or experimental co-crystal complexes. Complexes can be encoded as joint point clouds (ligand + pocket), bipartite interaction graphs, or spatial fields of contact/physicochemical signals. Topology may be computed on the ligand, the pocket/surface, or the complex, depending on whether the objective is recognition (pocket suitability), ranking (relative binding), or pose discrimination.

### From molecular data to topological objects

Most TDL workflows in drug discovery use PH, which requires choosing (i) a topological object and (ii) a filtration (an ordering across scales) [4, 5]. PH tracks births and deaths of features (components, loops, cavities) across the filtration, producing persistence diagrams/barcodes.

**Point sets, distance graphs, and complexes.** From 3D coordinates (atoms, residues, surface samples), one builds simplicial complexes from pairwise distances. Vietoris–Rips (VR) complexes are generic but can be expensive at scale. Geometry-aware alternatives such as Čech or alpha complexes can be more parsimonious in Euclidean settings [31]. In practice, drug-discovery pipelines typically control complexity by pocket localization, subsampling, or element-/channel-specific constructions.

**Surfaces and meshes.** Pockets and interfaces are often best described by surfaces. Triangle meshes or surface point clouds can be used directly for PH, and are particularly relevant for docking scoring where cavity/shape complementarity matters.

**Weighted graphs and higher-order constructions.** PH can also be computed on weighted graphs (ligand graphs, residue graphs, contact graphs) by thresholding edge weights. This route enables topology-aware summaries even without full 3D structure, but interpretability and stability depend on how weights are defined.

### Filtrations and signals: practical choices

The filtration determines what PH measures and is therefore the largest applied design degree of freedom. Useful filtrations for DTI/VS/docking usually encode either geometry or physicochemical/interaction signals.

**Distance-based filtrations.** Distance filtrations on point clouds yield VR/alpha complexes and provide a stable, task-agnostic starting point. Chemical specificity can be introduced by computing PH on element subsets or via type-aware distance constructions. Element-specific PH has been effective for binding-related prediction when paired with ML models [8].

**Sublevel filtrations on scalar fields.** When a scalar function is available on points/surfaces/volumes (e.g. electrostatics, hydrophobicity, distance-to-surface, docking-derived interaction energy), one can study sublevel-set topology. These filtrations are natural for interface “landscapes” in docking, but they are higher risk: they are sensitive to how the field is computed and can encode pipeline artifacts.

**Multichannel and task-aware constructions.** Because binding depends on multiple signals, practical approximations to multiparameter persistence often compute separate filtrations per channel and concatenate summaries. Such multichannel signatures have been used to improve screening/ranking at scale [18].

### Vectorizations and integration into learning pipelines

Persistence diagrams are not directly compatible with standard learners, motivating vectorizations and kernels. The applied trade-off is between stability, expressivity, and compute cost.

**Vectorizations and kernels.** Common vectorizations include persistence images (PIs), persistence landscapes (PLs), and Betti curves (BCs); kernels define positive-definite similarities directly on diagrams. A recent survey provides a systematic comparison of these options [15]. For drug-discovery workloads, vectorizations that batch cleanly and scale across many ligands/poses are typically preferred. The practical choice depends on the task and the downstream learner, and the three main options have distinct trade-offs.

PIs [32] discretize the birth–death plane into a fixed-resolution grid with a Gaussian kernel, producing a flat vector that feeds directly into any standard regressor or convolutional neural network (CNN). PIs are the default choice for **docking scoring and affinity regression**: they are differentiable (enabling end-to-end training), easy to concatenate across element-pair channels (as in ESPH pipelines [8]), and their resolution and bandwidth are readily ablated. The main cost is sensitivity to the grid resolution and bandwidth hyperparameters, which must be cross-validated.

PLs [33] are piecewise-linear summaries that lie in a Banach space, giving them stronger theoretical stability guarantees than PIs. PLs are well suited to **DTI and classification tasks** where statistical hypothesis testing or uncertainty quantification over a set of compounds is required, because the Banach-space structure supports bootstrap confidence intervals directly on the feature vectors. Their main disadvantage in high-throughput settings is that the number of landscape levels needed for expressive coverage can grow with the complexity of the diagram, making dimensionality control more manual than with PIs.

(BCs record, for each scale value, the count of active topological features in each homology dimension. BCs are a natural fit for **VS and ranking**, where fast extraction and low memory footprint matter more than fine-grained diagram resolution. They are less expressive than PIs or PLs but often competitive when combined with strong ligand-based features (ECFP, GNN embeddings), and their scalar-valued nature makes them trivial to concatenate and interpret.

*Diagram kernels* (e.g. the stable multi-scale kernel [34]) avoid explicit vectorization and work directly with kernel-based learners (SVMs, kernel ridge regression). They are theoretically principled but scale poorly to large datasets: computing the full Gram matrix over thousands of ligands or poses is prohibitive in high-throughput VS or docking scoring. Kernels are therefore most appropriate for **small-data DTI** settings where sample sizes are modest and computational cost is not the bottleneck.

As a practical default: use PIs for docking scoring and affinity regression; PLs when statistical guarantees over feature distributions matter; BCs for rapid prototyping or large-scale screening; and kernels only when data are scarce and a kernel-based learner is already in use. Regardless of choice, vectorization should always be ablated alongside the filtration (Rule 5, Box 2).

**Integration patterns.** Most applied TDL pipelines compute topological features per modality (ligand/pocket/complex) and fuse them with non-topological representations (e.g. GNN embeddings or sequence encoders) via early fusion (concatenation) or late fusion (ensembling/attention). Topological descriptors can be effective inputs to deep predictors [1], and broader TDL surveys summarize integration choices [2, 3].

**Practical guidance.** For DTI, start from strong ligand-only and target-only baselines, then add topology on the modality with the most reliable 3D signal (often the pocket). For SBVS and docking scoring, pocket/surface topology is frequently more robust than complex-level topology unless pose uncertainty is handled explicitly. When in doubt, prefer constructions that are stable, interpretable, and easy to ablate.

**Box 1: Glossary (minimal)**

- **Persistent homology (PH)** Multiscale descriptor tracking topological features across a filtration.
- **Filtration** Nested family of spaces/complexes indexed by a scale or threshold parameter.
- **Persistence diagram/barcode** Birth–death (diagram) or interval (barcode) summaries of PH.
- **VR/alpha complex** Distance-based (VR) versus geometry-aware (alpha) simplicial complexes built from point sets.
- **Vectorization** Map from diagrams to fixed-length features (e.g. images, landscapes, BCs).
- **Pocket/pose** Binding-site region (pocket) and a specific docked placement of a ligand (pose).
- **EF/BEDROC** Early-recognition metrics for VS emphasizing top-ranked actives.
- **Scaffold split** Train/test split separating compounds by core scaffolds to probe chemotype generalization.

## A task-driven taxonomy of TDL methods for DTI/VS/docking

**Figure 3:**
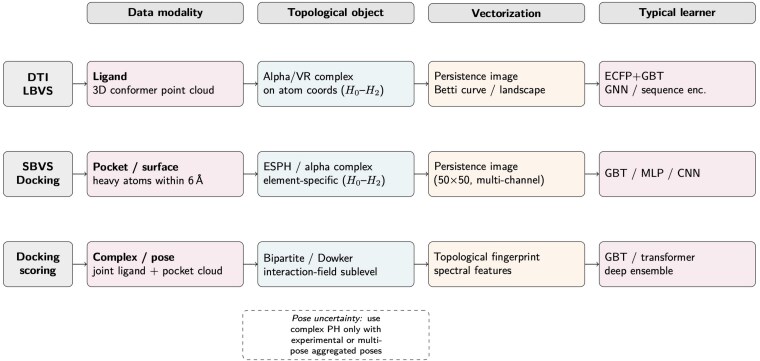
From data modalities to topological representations and learners (Sec. 2).

**Figure 4:**
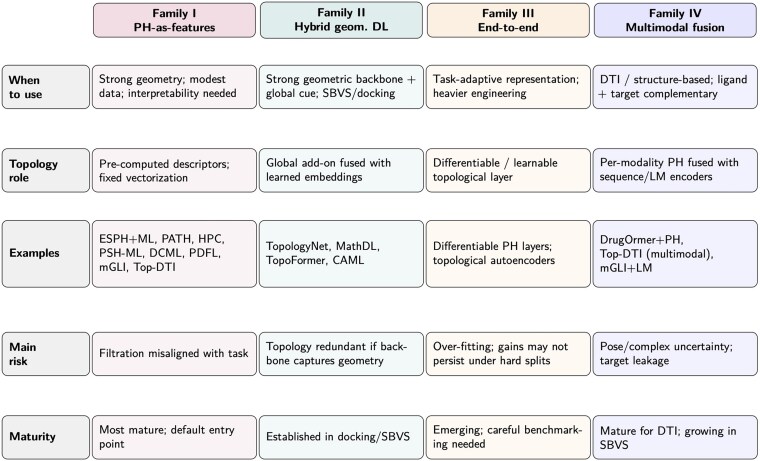
Comparative overview of the four TDL method families (Section A task-driven taxonomy of TDL methods for DTI/VS/docking).

Section Data modalities and topological representations for DTI/VS/docking described the main design choices in TDL pipelines: modality (ligand/target/pocket/complex), topological construction (object + filtration), and vectorization/integration. Here we organize the applied literature into four pragmatic families aligned with common workflows in DTI, VS, and docking scoring. The goal is operational: what to implement first, what to ablate, and which failure modes typically appear under hard splits. Figure 3 illustrates the mapping from data modalities to topological objects and vectorizations for the three tasks. Figure 4 provides a comparative overview of the four families.

### Family I: PH-as-features (topology as descriptors)

**When to use.** PH-as-features is the default entry point because it is modular, ablatable, and comparatively mature. It is most suitable when (i) the signal is strongly geometric (3D conformers, pockets/surfaces, interfaces), (ii) sample size is modest relative to model capacity, or (iii) interpretability is a requirement. It has been effective in early topology-informed biomolecular predictors [1] and in molecular PH representations designed for downstream learning [7].

**Typical pipeline.** A standard workflow is as follows: (i) define the object (ligand conformer point cloud, pocket/surface samples, or complex/interface points); (ii) choose a filtration (usually distance/alpha; optionally element-specific or multichannel); (iii) compute persistence diagrams in low dimensions (commonly $H_{0}$–$H_{2}$); and (iv) vectorize for learning. Widely used vectorizations include PLs [33] and PIs [32]. Alternatively, diagram kernels avoid explicit vectorization; stable multi-scale kernels are a classical choice [34]. In applications, topological features are typically concatenated with standard descriptors (ECFP, GNN embeddings, sequence encoders) or used as a separate branch in a multimodal model. Representative Family I methods from Table 1 include ESPH+ML [8], which applies element-specific distance filtrations on protein–ligand complexes and feeds vectorized persistence summaries into gradient-boosted or shallow-network regressors for affinity prediction; PATH [21], which uses persistence fingerprints from interaction geometry combined with gradient-boosted trees for binder/non-binder scoring; and HPC [22], PSH-ML [23], PerSpect ML [24], DCML [25], and PDFL [27], which explore cohomology, spectral, Dowker-complex, and directed-flag Laplacian constructions, respectively, all feeding fixed-length descriptors into standard ML regressors. For VS, PH VS [17] and ToDD [18] apply multiparameter persistence and topological fingerprints to ligand conformers for ligand-based ranking. For DTI, Top-DTI [10] uses PH summaries derived from ligand–protein contact graphs as features for interaction prediction.

**Strengths and limits.** The main advantages are clean ablations (topology on/off without altering the backbone), reusable feature extractors across tasks, and features that can often be related back to multiscale structural motifs. The main costs are compute and engineering around PH extraction: VR complexes can become expensive, while alpha complexes are often more parsimonious in Euclidean settings [31]. Practical implementations therefore control point counts (pocket localization, subsampling), restrict homology dimensions, and cache PH outputs.

**Common failure modes.** PH-as-features degrades when the filtration is misaligned with the task (“measuring the wrong structure”), when conformer/pose uncertainty dominates (notably in docking scoring), or when the downstream learner is weak compared with strong non-topological baselines. A recurring pitfall is reporting improvements under random splits that vanish under scaffold or target-wise/family splits; minimum standards are specified in Section Benchmarks, evaluation protocols, and reproducibility.

### Family II: hybrid geometric DL with topological signals

**When to use.** Hybrid methods add topology as a complementary *global* cue on top of strong geometric baselines (2D GNNs, residue graphs, 3D equivariant models). They are most common in SBVS and docking scoring, where pocket/interface organization is multiscale and not always recovered by local message passing.

**Where topology enters.** Two integration patterns dominate: *early fusion* (concatenate PH features with learned embeddings) and *late fusion* (ensembling or gated/attention merging of topology and geometry branches). Early fusion supports clearer attribution; late fusion can be more robust when pose/pocket variability causes modality-specific failures. Representative Family II methods include TopologyNet (TNet-BP) [1], which combines element-specific PH channels with a CNN/MLP scorer for docking affinity; MathDL (GC2/GC3 and GC4) [9, 20], which fuses multiscale algebraic graphs with ESPH topological fingerprints in deep ensemble architectures for pose selection and affinity ranking; and TopoFormer [12], which converts protein–ligand 3D structure into topological sequences via a persistent hyperdigraph Laplacian and fine-tunes a transformer for scoring, ranking, docking, and screening tasks. CAML [13] occupies a Family I/II boundary, combining commutative-algebra descriptors with ML regressors on PDBbind and CASF benchmarks.

**Practical cautions.** Topology can be redundant if the backbone already captures the relevant geometry, or noisy if pocket extraction or pose generation is unstable. Accordingly, studies should report topology on/off under leakage-resistant splits and include at least one fusion ablation (early versus late fusion or single-branch versus fused).

### Family III: end-to-end/differentiable topology (when applicable)

**When to use.** End-to-end topology is attractive when one expects the representation (or topological inductive bias) to be task-adaptive and learnable. In DTI/VS/docking, these approaches remain less standardized than PH-as-features and often require heavier engineering and careful benchmarking.

**Representative mechanisms and maturity.** Representative directions include differentiable topological signatures inside deep models [35] and latent-space regularization that preserves prescribed topological properties [36]. TopoFormer [12] pushes toward this family through its self-supervised MAE pretraining on topological sequences, where the topological representation is learned end-to-end alongside the transformer rather than precomputed and frozen. Given the added degrees of freedom, gains should be treated as *emerging* unless they persist under hard splits and against capacity-matched baselines [2, 3].

### Family IV: multimodal fusion for ligand–target systems

**When to use.** Multimodal fusion is the default for DTI and structure-based pipelines because ligand chemistry and target information are complementary. Topology can be computed per modality (ligand/pocket/complex) and fused alongside learned encoders, or computed on the complex when poses are reliable.

**Fusion patterns and pose uncertainty.** At scale, ligand + protein (sequence/LM) fusion is common; when structures exist, pocket-first topology provides a pose-free option that avoids docking noise. For docking scoring, poses should be treated as a set (multi-instance aggregation) and sensitivity to pose generation should be reported. When pose quality is unstable, pocket-only or pocket+ligand (pose-free) designs are usually preferable. Recent examples combine strong multimodal baselines with topology as a complementary signal [10, 11]. The mGLI/KDA framework [14] exemplifies a Family IV-compatible design: multiscale Gauss link integral features from atomic curve structures are paired with sequence-based language-model embeddings and fed into gradient-boosted or deep-network regressors, achieving competitive performance across 13 biological datasets including protein–ligand affinity and toxicity screening.

### TDL versus equivariant geometric deep learning: complementarity and limits

A recurring question for practitioners is when TDL adds value over modern 3D-equivariant models such as SchNet [37], PaiNN [38], DimeNet [39], NequIP [40], or MACE [41], which encode atomic geometry with SE(3)- or E(3)-equivariant message passing and have achieved strong results on molecular property prediction benchmarks. The answer is not “one replaces the other” but rather that the two approaches capture different aspects of 3D molecular structure, which is why hybrid designs (Family II) are the most competitive in drug-discovery settings.

**What equivariant models capture well.** Equivariant GNNs encode *local geometry* with high fidelity: interatomic distances, bond angles, dihedral torsions, and direction-dependent interactions are processed through tensor-valued message passing that respects rotation and reflection symmetry by construction. This makes them highly data-efficient for learning smooth potential-energy surfaces and force fields, where small coordinate perturbations must map to consistent energy changes. Their inductive bias directly encodes the physical symmetries of the problem, which is a strong prior for quantum-chemical and conformational tasks.

**What PH captures that equivariant models do not.** PH extracts *global and multiscale topological invariants*—connected components, loops, and cavities—that emerge only when aggregating information across the entire structure or across a range of distance scales simultaneously. A local message-passing network, however expressive or deep, cannot recover these global invariants without an architecture explicitly designed to do so: a binding pocket channel that only becomes apparent at a scale larger than the message-passing radius is invisible to a standard EGNN operating with a fixed cutoff. PH is also invariant to small coordinate perturbations by the stability theorem for persistence diagrams [42], a different kind of robustness from equivariance—it is robust to geometric noise rather than to rigid transformations. Finally, PH features are discrete and combinatorial, making them straightforward to inspect, ablate, and map back to structural motifs; this interpretability is uneven in deep equivariant networks where attribution requires additional tools.

**What PH does not capture.** Topology discards orientation and metric precision: two structures with the same persistence diagram can differ substantially in bond lengths, angles, and chirality. Element-specific constructions (ESPH [8]) partially address chemical specificity, but PH remains insensitive to directional features such as hydrogen-bond geometry or dihedral preferences that equivariant models encode naturally. PH also requires a choice of filtration and complex that is not learned from data in the Family I setting, introducing a manual design step that equivariant models avoid.

**Practical implication: complementarity, not substitution.** The two approaches are most naturally combined: equivariant backbones handle local geometry and physical symmetries, while PH contributes global and multiscale cavity/loop signals that local message passing does not recover. This is the rationale for Family II in this review, and it is validated empirically by methods such as TopologyNet [1], MathDL [9], and TopoFormer [12], which achieve competitive performance by pairing topological features with learned geometric representations. The practical design question is therefore not “PH or equivariant GNN” but rather: *does the task involve global pocket/surface structure that local message passing misses?* If yes, PH is a principled and interpretable complement; if the task is dominated by local bond geometry or requires continuous force predictions, equivariant models alone may suffice.

**Box 2: Design rules of thumb**

- **Rule 1 (start simple).** Begin with strong non-topological baselines, then add PH as a modular component.
- **Rule 2 (align filtration).** Use distance/alpha for shape; use field-based sublevel filtrations only with controls.
- **Rule 3 (evaluate like deployment).** Prefer scaffold + target-wise/family/cluster splits over random splits.
- **Rule 4 (report uncertainty and compute).** Report seeds/CIs, PH settings, and conformer/pose counts.
- **Rule 5 (ablate topology).** Topology on/off plus filtration/vectorization variants are minimum requirements.

## Applications across the pipeline: DTI, VS, docking scoring

DTI prediction, VS, and docking scoring occupy different parts of the discovery pipeline and fail for different reasons. DTI is dominated by label noise, missing negatives, and cold-start generalization (new targets and chemotypes). VS is an early-recognition ranking problem in which dataset bias and protocol choices can dominate apparent gains. Docking scoring is structure-conditioned prediction where pose uncertainty and target leakage often overwhelm architectural novelty. Across all three, topology is most useful when it adds a stable, multiscale geometric signal that is complementary to strong baselines.

### Drug–target interaction

#### Problem formulations and data availability

DTI appears as (i) binary interaction classification (or thresholded activity), (ii) multiclass outcome prediction, or (iii) affinity regression. This choice is not cosmetic: it determines label construction, negatives, and what constitutes out-of-distribution generalization.

**Data sources, heterogeneity, and negatives.** Large-scale DTI corpora are typically assembled from BindingDB and ChEMBL [43, 44], with drug/target annotations from resources such as DrugBank [45]. Because measurements span heterogeneous assays and conditions, curation and unit normalization (e.g. to p$K_{d}$/p$K_{i}$/pIC$_{50}$) often determine practical performance. For classification, the key modeling degree of freedom is the *negatives policy*: unobserved interactions are not reliable negatives, and decoy-style negatives are uncommon in DTI. Claims of topological benefit should therefore be reported under at least one alternative negatives construction whenever feasible.

**Benchmarks and cold-start regimes.** Historically standard affinity benchmarks (Davis, KIBA) [46, 47] remain useful for comparability and iteration speed, including in DeepDTA-style baselines [48], but they probe a narrow target family and can overstate generality. Whenever possible, complement them with broader repository-derived subsets and standardized tooling (e.g. TDC) that supports split protocols designed for generalization [49]. In practice, the most relevant settings are cold-target and cold-chemotype evaluation, where topology can plausibly help by capturing pocket/surface geometry that is hard to infer from local representations alone.

#### Where topology enters (ligand, pocket, complex)

**Ligand topology (conformer-controlled).** If conformers are standardized, ligand PH can summarize multiscale shape and ring/cavity patterns. In DTI, this is mainly useful for scaffold generalization, but it remains limited because binding is relational and conformer uncertainty can dominate.

**Pocket/surface topology (structure-aware, pose-free).** Pocket-level topology is often the highest-value insertion point when structures are available. Pockets exhibit multiscale cavities and channels linked to recognition and selectivity, and element-/channel-specific PH can yield informative descriptors for binding endpoints [8]. Engineering-wise, pocket PH reduces reliance on docking and is therefore less sensitive to pose noise.

**Complex-level topology (pose-sensitive).** Complex PH can encode interface complementarity, but is credible only when poses are experimental, standardized, or aggregated across multiple poses. Otherwise, complex topology risks learning artifacts of the docking pipeline. A pragmatic default is pocket topology fused with ligand features (cf. Families II/IV in Section A task-driven taxonomy of TDL methods for DTI/VS/docking), reserving complex PH for controlled-pose settings.

#### Representative results and practical takeaways

A consistent empirical pattern is that topology adds the most value under *hard splits* (scaffold and target-wise/family splits), where leakage and near-duplicate similarity are suppressed. In competitive systems, topology is typically treated as a complementary branch fused with strong ligand (ECFP/GNN) and protein (sequence/LM) encoders rather than as a replacement. Recent DTI-focused examples follow this multimodal pattern [10, 11].

**Practical takeaways.** Implement pocket topology first when reliable structures exist; prefer PH-as-features or simple hybrid fusion before end-to-end topology; and treat scaffold + target-wise/family splits as non-negotiable for generalization claims.

**What to report (DTI).**

- Dataset(s) and exact curation; negatives policy; version/date (e.g. BindingDB/ChEMBL snapshot) [43, 44].
- Split protocol: scaffold split and target-wise/family split (state thresholds and clustering procedure).
- Metrics: ROC-AUC and PR-AUC (classification) and RMSE/MAE + Spearman (regression), with uncertainty.
- Baselines: ligand-only, protein-only, and a standard multimodal model without topology (DeepDTA-style or stronger) [48].
- Ablations: topology off/on; filtration/vectorization sensitivity; conformer count/protocol if used.

### Virtual screening

#### Ligand-based versus structure-based VS

VS is a ranking task: the objective is to retrieve actives early in a large list. Distinguish ligand-based VS (LBVS; ligand-only similarity/prediction) from structure-based VS (SBVS; pocket/surface-conditional ranking, often with docking). Topology is most naturally aligned with SBVS because pocket geometry and surface cavities are multiscale and global.

#### Where topology helps (pockets, surfaces, ensembles)

For SBVS, pocket/surface PH can act as a stable global descriptor that complements local geometric DL, particularly when pocket definition varies across structures. Multichannel or multiparameter topological signatures have also been used for topology-aware representation learning in screening [18]. A second use case is ensemble robustness: compute PH per ligand conformer or per docked pose and aggregate (e.g. pooling/attention) to reduce sensitivity to a single generated structure. Such designs should be accompanied by sensitivity analyses over conformer/pose generation.

#### Bias, leakage, and metrics

VS evaluation is unusually sensitive to benchmark design and redundancy. DUD-E and DEKOIS 2.0 are widely used docking benchmarks [50, 51], while LIT-PCBA was proposed to reduce bias and improve realism [52]; recent audits highlight that leakage/redundancy can still arise from how these benchmarks are processed and split [53]. For topology-aware SBVS, target similarity is an additional leakage path: pocket topology can act as an identifier if homologous targets (or near-identical pockets) appear across splits. Finally, metrics must reflect early recognition: EF and BEDROC were designed to emphasize performance at the top of the ranked list [54].

**Practical takeaways.** For SBVS, prefer pocket/surface topology over complex/pose topology unless pose uncertainty is explicitly controlled. For LBVS, treat ligand topology as optional and justify it with conformer standardization and aggregation. In both regimes, protocol choices (splits, redundancy removal, decoy assumptions) often dominate the apparent effect size.

**What to report (VS).**

- Dataset(s) and decoy assumptions; preprocessing and redundancy removal [50–52].
- Split protocol: scaffold/temporal; for SBVS add target-wise/cluster splits (state identity/site-similarity criteria).
- Metrics: EF@1%, EF@5%, BEDROC (+ ROC-AUC if included) [54].
- Baselines: ECFP+ML (LBVS) and at least one strong SBVS/3D baseline without topology.
- Ablations: topology off/on; aggregation/sensitivity to conformer/pose generation.

### Docking scoring

#### Task scope: pose selection versus scoring/ranking

Docking-related learning tasks should be separated. *Pose selection* (docking power) chooses a near-native pose among candidates, while *scoring/ranking* predicts affinity or ranks ligands. This review prioritizes scoring/ranking, treating pose selection only as a source of noise that can confound learning.

#### Topology at the pocket or at the interface

Docking scoring is geometrically driven, so topology can be meaningful, but the insertion point matters.

**Pocket-first (pose-free) topology.** Compute PH on pocket atoms or surface samples (optionally with physicochemical channels) and fuse it with ligand features. This design is robust to docking noise and is a strong default when poses are unreliable.

**Complex/pose-level topology (controlled poses only).** Complex PH on the joint ligand–pocket point cloud (or on interaction-derived scalar fields) can encode interface complementarity. However, it is highly sensitive to pose uncertainty and can amplify systematic docking artifacts. Complex-level topology is therefore most credible with experimental poses, standardized docking protocols, or explicit multi-pose aggregation with sensitivity reporting.

#### Generalization and leakage control

Docking scoring benchmarks are commonly built from PDBbind [55] and are often summarized using CASF-style evaluations [56]. For topology-based approaches, the dominant risk is target leakage: homologous proteins, similar pockets, or overlapping ligand series can appear across splits. Target-cluster splits and explicit leakage checks should therefore be treated as default requirements.

**Practical takeaways.** Start with pocket-first topology; add complex topology only when pose uncertainty is managed; and prioritize target-cluster splits with documented leakage controls.

**What to report (Docking scoring).**

- Dataset(s) (affinity versus pose labels) and pose generation/selection protocol (PDBbind/CASF family) [55, 56].
- Split protocol: target-cluster split (state thresholds); leakage controls for homologs and ligand series.
- Metrics: affinity (RMSE/MAE + Spearman) and/or pose success (top-$1$/top-$N$ RMSD thresholds) if pose selection is included.
- Baselines: classical scoring/feature baseline + one geometric DL baseline without topology.
- Ablations: topology off/on; sensitivity to pose generation and aggregation when complex topology is used.

### An illustrative end-to-end example

To anchor the taxonomy and reporting requirements in practice, we trace a concrete TDL workflow for docking scoring (Family I, PH-as-features) through the full pipeline described in Sections Data modalities and topological representations for DTI/VS/docking and A task-driven taxonomy of TDL methods for DTI/VS/docking.

**Dataset and curation.** Start from the PDBbind refined set; remove complexes with resolution ${>}2.5$ Å and nonstandard residues; record the dataset version and curation date.

**Topological construction.** Extract binding pockets defined as all heavy atoms within 6 Å of the co-crystallized ligand. Compute element-specific alpha-complex persistent homology (ESPH [8]) in dimensions $H_{0}$–$H_{2}$ on C, N, O, S atom subsets; restrict to pairwise interaction cutoffs of 4–12 Å to control computational cost.

**Vectorization.** Map each persistence diagram to a PI (resolution $50{\times }50$, Gaussian kernel, bandwidth selected by cross-validation) and concatenate across element-pair channels, yielding a fixed-length feature vector per complex.

**Learning and evaluation.** Train a gradient-boosted regressor (e.g. XGBoost) to predict p$K_{d}$. Evaluate on the CASF-2016 core set [56] using a target-cluster split (30% sequence-identity threshold); report Pearson $r$, RMSE, and Spearman $\rho$ with bootstrap 95% confidence intervals over at least three random seeds.

**Baselines and ablations.** Compare against (i) a topology-off baseline using the same regressor on ECFP+pocket physicochemical features, and (ii) a classical docking score baseline. Report the topology on/off $\Delta$ with paired bootstrap confidence intervals. Additionally, ablate the filtration (distance-only versus ESPH) and the vectorization (PI versus BC) to isolate where the topological gain originates.

This five-step walkthrough instantiates the design rules of Box 2 and the minimum reporting standards of Section Benchmarks, evaluation protocols, and reproducibility, and can be adapted to VS (replacing the affinity target with EF/BEDROC and the split with a scaffold or target-wise protocol) or to DTI (replacing the pocket-level construction with ligand or sequence features as appropriate).

## Benchmarks, evaluation protocols, and reproducibility

This section is an evaluation playbook for TDL in DTI, VS, and docking scoring. In these tasks, headline gains are often dominated by (i) dataset construction/curation, (ii) split design and leakage control, and (iii) metric choice (especially early-recognition metrics for VS). Accordingly, topology should be considered compelling only if it is validated under deployment-relevant splits, against strong baselines, with uncertainty reporting and transparent compute/tuning budgets.

Table 2 summarizes recommended default datasets, splits, metrics, and common pitfalls for DTI, VS, and docking scoring.

### Benchmark selection criteria by task

Benchmark choice should be driven by the *deployment question* rather than by tradition. Below we emphasize practical suitability criteria and the minimum limitations that should be stated.

**DTI (classification/regression).** DTI benchmarks typically fall into (i) narrow, historically standard kinase-focused affinity sets (e.g. Davis, KIBA) [46, 47], which are useful for comparability and iteration speed but overstate generality; and (ii) broader repository-derived subsets (e.g. BindingDB/ChEMBL curation) [43, 44], which better reflect assay heterogeneity and target/chemotype diversity. A DTI benchmark is suitable if it documents assay/unit normalization, specifies the negatives policy (implicit versus curated negatives), reports redundancy control, and supports hard splits (scaffold and protein-family/target-wise). When possible, prefer standardized tooling that fixes task definitions and split generators (e.g. TDC) [49].

**Virtual screening (ligand-based VS versus SBVS).** VS is a *ranking* problem, so benchmark suitability hinges on (i) decoy assumptions, (ii) analog-bias controls, (iii) target leakage controls (for SBVS), and (iv) availability of early-recognition evaluation. DUD-E and DEKOIS 2.0 remain common docking benchmarks [50, 51], while LIT-PCBA was introduced to reduce biases that can inflate ML performance [52]. However, benchmarks remain sensitive to redundancy and leakage, and audits show that preprocessing choices can reintroduce leakage even in “improved” settings [53]. For BiB-level claims, it is insufficient to state only the benchmark name: report the exact preprocessing, redundancy removal, and split protocol.

**Docking scoring (affinity scoring versus pose selection).** Docking-related evaluation must separate: (i) scoring/ranking (typically PDBbind-derived) [55] and (ii) pose selection (RMSD-based docking-power protocols). This review prioritizes scoring/ranking, but pose noise can contaminate scoring evaluation. CASF-style benchmarks were designed to separate scoring, ranking, and docking power; CASF-2016 is a standard reference [56]. A suitable docking-scoring benchmark should specify the pose-generation protocol, target redundancy controls, and whether evaluation is within-target or cross-target.

### Splits and leakage: minimum standards

In DTI/VS/docking, the split protocol is often the decisive factor: models that look strong under random splits can fail under realistic generalization. We recommend reporting *at least one hard split* aligned with intended deployment.

**Scaffold splits (ligand generalization).** Scaffold splits probe chemotype generalization by grouping compounds via Bemis–Murcko scaffolds and splitting at the scaffold level [57]. They are essential for DTI and VS. Report: scaffold definition tool (e.g. RDKit), handling of rare scaffolds, and seed(s).

**Temporal splits (prospective realism).** If timestamps exist, temporal splits (train on earlier data, test on later data) reduce overoptimism caused by repeated measurement of related series. Report the time cutoff and include a brief discussion of dataset drift.

**Protein-family/target-wise splits (target generalization).** To test generalization to unseen targets, cluster proteins by sequence identity (or family annotation) and split by clusters. State the identity threshold (e.g. 30% or 40%) and clustering procedure. For SBVS and docking scoring, also consider *binding site similarity*: distinct sequences can share near-identical pockets, which can leak pocket identity and inflate pocket-topology methods.

**Task-specific leakage checks.** Minimum checks include (i) **DTI:** remove duplicate (ligand, target, label) records; deduplicate compounds by canonical SMILES; avoid splitting replicates of the same pair across train/test unless explicitly modeling replicate noise. (ii) **VS:** ensure decoys do not appear as actives elsewhere; control analog bias via scaffold splits; for SBVS, apply target-wise/cluster splits so that near-identical pockets are not shared across splits. (iii) **Docking scoring:** control ligand-series leakage (same scaffold families across splits) and target/pocket leakage (homologs, same pocket). If multiple poses per complex are used, avoid training on poses generated from the same crystal complex used for testing unless the evaluation is explicitly within-target.

### Metrics and uncertainty reporting

Metrics must match the objective and should be accompanied by uncertainty estimates. We recommend reporting uncertainty across both *data resampling* and *training randomness* (seeds).

**DTI.** For classification: report ROC-AUC and PR-AUC; PR-AUC is often more informative under class imbalance. For regression: report RMSE/MAE and rank correlations (Spearman; optionally Kendall). ROC-AUC confidence intervals can be obtained by DeLong’s method [58] or stratified bootstrap; for regression/ranking, use paired bootstrap over test items.

**VS.** VS should emphasize early recognition: report EF@1%, EF@5%, and BEDROC [54] (ROC-AUC only as secondary). If EF/BEDROC are omitted, justify why the application does not require early recognition.

**Docking scoring.** For affinity scoring: RMSE/MAE and Spearman are common. For pose selection (if included): report top-$1$/top-$N$ success under RMSD thresholds (e.g. 2Å) and specify heavy-atom versus all-atom RMSD and symmetry handling.

**Recommended uncertainty defaults.**

- **Bootstrap CIs:** stratified bootstrap (classification) or paired bootstrap (ranking/regression), with at least 1000 resamples.
- **Across-seed reporting:** mean $\pm$ standard deviation over at least three seeds; ideally five seeds when compute permits.
- **Paired comparisons:** use paired resampling where possible and report effect sizes (e.g. median improvement) in addition to $P$-values.

### Baselines and ablation studies

TDL methods must be compared against strong non-topological baselines and must isolate where improvements originate.

**Mandatory baselines (by task).**

- **DTI:** ligand-only baseline (e.g. ECFP + RF/XGBoost), protein-only baseline (sequence encoder), and a standard multimodal baseline without topology (e.g. DeepDTA-like or modern graph+sequence) [48].
- **VS:** ECFP similarity or ECFP+ML baseline (ligand-based), and at least one strong 3D/SBVS baseline when structure is used.
- **Docking scoring:** a classical docking-score baseline and one geometric DL baseline without topology.

**Ablations that isolate topology.** At minimum: (i) topology off versus on with the same backbone; (ii) filtration variants (distance-only versus physchem/interaction); (iii) vectorization variants (image versus landscape versus BC); and (iv) sensitivity to key parameters (points/atoms, homology dimensions, max filtration).

**Compute and tuning transparency.** Because topology can change compute costs, report: GPU type and total GPU-hours, PH library/settings, conformer/pose counts, and hyperparameter search budget. If topology gains require substantially larger tuning budgets, state this explicitly.

**Checklist 1: Minimum reporting standard for TDL in DTI/VS/docking**

- Data curation and versions documented; exclusions and unit normalization stated.
- Split protocol fully specified (including seeds), with leakage controls and redundancy checks.
- Metrics aligned to task (include EF@1%, EF@5%, BEDROC for VS).
- Uncertainty reported (bootstrap CIs and/or across-seed variability).
- Baselines include at least one strong non-topological model of similar capacity.
- Ablations isolate topology (off/on; filtration and vectorization variants).
- Compute and tuning budgets reported (including PH settings and conformer/pose counts).
- Code/configuration shared when possible (or limitations clearly stated).

## Limitations and open challenges

TDL for DTI/VS/docking is promising, but robust gains are still constrained by three coupled factors: (i) molecular data and label quality, (ii) geometric uncertainty (conformers/poses and pocket definition), and (iii) evaluation discipline (splits, leakage control, and metric alignment). Below we summarize the main bottlenecks and the minimum robustness checks that should accompany any topology-based performance claim.

### Scalability and computational bottlenecks

PH can become a practical bottleneck when applied to large point clouds or to large ensembles (many ligands, conformers, or poses). Cost depends strongly on the complex choice (e.g. VR versus alpha) and on the maximum homology dimension [31]. In high-throughput settings, computing PH on full atomic clouds for every generated pose is rarely viable without aggressive controls.

In practice, most DTI/VS/docking signal is captured in low dimensions (0–2) with strict point-count management (pocket localization, subsampling, or element-/channel-specific subsets). PH-as-features pipelines (Section Family I: PH-as-features (topology as descriptors)) remain the most compute-predictable because topology extraction is decoupled from training and can be cached. Fixed-size vectorizations (images/landscapes/BCs) simplify batching and deployment [15]. The main open scaling challenge is *ensemble scaling*: realistic SBVS and docking scoring often require multiple poses per ligand and sometimes multiple protein conformations. Efficient multi-instance aggregation that preserves topological signal without exploding cost remains underdeveloped.

### Sensitivity to filtrations and hyperparameters

Filtration design is the largest source of silent degrees of freedom: a method may appear to work because the filtration encodes target identity, conformer-generation artifacts, or docking protocol quirks, rather than transferable binding signal. Conversely, topology can appear ineffective if the filtration is misaligned with the task.

Minimum robustness checks should therefore include (i) topology off/on with a fixed backbone, (ii) at least one alternative filtration family (e.g. distance-only versus a physchem/interaction signal), and (iii) sensitivity to key parameters (point count, homology dimensions, max filtration). Vectorization should also be ablated because stability/expressivity differ across representations [15]. For conformers/poses, report performance versus the number of conformers/poses and the generation protocol; for pocket extraction, state the rule (radius/residue selection/surface settings) and test at least one reasonable alternative.

### Generalization under distribution shift

Distribution shift is the dominant failure mode in drug discovery ML: new target families, new chemotypes, new structures, altered binding-site conformations, and changed docking protocols can all break apparent in-distribution gains. Random splits largely test interpolation; they do not establish deployable performance. Accordingly, scaffold and target-wise/family splits are minimum standards for DTI and SBVS, and target-cluster splits are essential for docking scoring (Section Benchmarks, evaluation protocols, and reproducibility). When topology is computed on pockets or complexes, target leakage risk increases because geometric signatures can act as identifiers; generalization claims should therefore be read primarily through hard splits and explicit leakage controls.

### Interpretability and mechanistic insight

Interpretability is often cited as a motivation for topology (cavities/channels/multiscale motifs), but it is uneven across method families. PH-as-features is typically the most interpretable because features can be inspected and ablated, especially with localized constructions (pocket/surface rather than whole protein) and element-/channel-specific filtrations. For hybrid and end-to-end approaches, attribution becomes harder because fusion mechanisms can entangle topological and non-topological signals, and improvements may lack a clear structural explanation [2, 3]. Standardized interpretability protocols (and consistent reporting) remain an open need.

### Protein flexibility and conformational ensembles

A recurring practical limitation of static TDL pipelines is that proteins are not rigid: binding pockets can undergo conformational changes upon ligand binding (induced fit), and the same target may adopt distinct conformations relevant to different ligand chemotypes. Ignoring this source of variability can inflate or deflate apparent TDL contributions, particularly for SBVS and docking scoring where pocket topology is the primary signal.

Several strategies can partially address this within TDL workflows. First, *pocket-ensemble topology*: when multiple crystal structures or MD snapshots are available, PH can be computed per conformation and summarized via pooling (mean/max over persistence diagrams or their vectorizations) or attention-based aggregation. This is analogous to multi-pose aggregation for ligands (Section Docking scoring) and carries similar requirements: sensitivity to the number and selection of conformations should be reported. Second, *coarse flexibility descriptors* (e.g. B-factors, crystallographic disorder, or NMA-derived modes) can be incorporated as scalar channels in filtrations, allowing PH to partially encode conformational uncertainty at the filtration stage rather than through explicit ensembles. Third, when structures are unavailable, *pocket prediction confidence* (e.g. AlphaFold2 pLDDT scores) should be treated as additional uncertainty to report, since topology computed on low-confidence regions carries less mechanistic meaning.

In practice, the minimum standard for any TDL study using static structures is to (i) state the source and resolution of structures used, (ii) note whether known alternative conformations exist for the targets tested, and (iii) test robustness to at least one structural variant (e.g. apo versus holo, or two crystal forms) when feasible. The mGLI framework [14] and related multiscale knot data analysis approaches demonstrate that topology can be computed on curve-like molecular structures—potentially including backbone traces and loop geometries—opening a path toward representing local conformational flexibility topologically.

### When *not* to use TDL

Topology is not a universal improvement lever. Prefer not to use TDL (or use it only as exploratory) when:

- **Geometry is not central or not controllable:** 3D conformers/poses are unreliable and cannot be standardized.
- **Evaluation cannot control bias/leakage:** scaffold/target-wise splits and redundancy checks are not feasible, making geometry-derived gains hard to trust.
- **Compute is the binding constraint:** PH over large pose/conformer ensembles is prohibitive; lightweight baselines or pocket-first descriptors are preferable.

In summary, topology is most effective when treated as a disciplined, ablatable signal rather than as a blanket replacement for geometric DL. The next section distills these lessons into actionable recommendations.

## Practical recommendations and outlook

R1. **Match topology to the modality you can control.** Start from strong non-topological baselines and add topology only where the 3D signal is reliable and standardized. For DTI, combine ligand baselines (ECFP/GNN) with protein sequence or LM encoders, and add topology primarily on the *pocket/surface* when structures are available. For VS, separate ligand-based VS from SBVS: in SBVS, pocket/surface topology is the default; in ligand-based VS, use ligand topology mainly when conformer ensembles are generated with an explicit protocol and aggregation. For docking scoring, prefer pocket-first (pose-free) topology unless pose uncertainty is explicitly handled (experimental poses or multi-pose aggregation with sensitivity analysis).
R2. **Choose filtrations that encode the intended signal.** Use distance/alpha filtrations as a robust first choice for multiscale shape, keep homology dimensions low (0–2), and control point counts via localization and subsampling. Use sublevel filtrations on physchem/interaction fields only with explicit controls, since these can inadvertently encode docking artifacts or target identifiers. In early deployments, prioritize parsimonious constructions and stable, fixed-size vectorizations (PIs, landscapes, BCs) to ease batching, ablations, and reproducibility.
R3. **Evaluate like deployment.** Treat split design as part of the method: random splits mainly test interpolation. DTI: report scaffold splits *and* target-wise/protein-family splits; use ROC-AUC/PR-AUC (classification) and RMSE/MAE plus rank correlation (regression). VS: emphasize early-recognition (EF@1%, EF@5%, BEDROC), and for SBVS add target-wise/cluster splits with explicit pocket/target leakage controls. Docking scoring: default to target-cluster splits (plus ligand-series leakage checks), report affinity metrics, and include RMSD success only when pose selection is in scope. Always include a capacity-matched non-topological baseline and a topology-off ablation.
R4. **Make topology gains falsifiable.** Report topology on/off, at least one filtration alternative, and at least one vectorization alternative. Provide sensitivity to point count, max filtration, and homology dimensions. When conformers/poses are used, report performance versus the number of conformers/poses and the generation protocol; treat improvements that vanish under modest perturbations as fragile and frame them accordingly.
R5. **Make reproducibility part of the method.** Document dataset snapshots and curation (including negatives policies), split definitions (seeds, thresholds, clustering), PH settings (complex type, dimensions, thresholds), conformer/pose parameters, and compute/tuning budgets. Share code and configuration when possible (or state limitations clearly) and summarize compliance with Checklist 1 to enable end-to-end replication and fair comparison.

**Key Points**

- Topological signals help most when molecular recognition is governed by multiscale 3D geometry (pockets, surfaces, interfaces) and when conformers/poses are noisy but controllable.
- Choose the modality you can standardize: pocket/surface topology for SBVS and docking scoring; ligand-conformer topology only with controlled conformer generation; complex-level topology only when pose uncertainty is handled (multi-pose aggregation or experimental poses).
- Evaluate “like deployment”: report at least one hard split (scaffold and target-wise/cluster), use early-recognition metrics for VS (EF@1%, EF@5%, BEDROC), and compare against strong non-topological baselines plus topology-off ablations.
- Minimum reproducibility standard: document dataset versions and curation (including negatives policy), split seeds/thresholds, PH settings (complex, dimensions, thresholds), compute and tuning budgets, and share code/configuration when possible.
- This review includes an end-to-end pipeline schematic (Fig. 1), a decision tree (Fig. 2), a curated methods matrix (Table 1), and a benchmarking playbook (Table 2 and Section Benchmarks, evaluation protocols, and reproducibility) to support implementation and fair comparison.

## Data Availability

No new data were generated or analyzed in support of this research.
